# The Multiple Faces of Nitric Oxide in Chronic Granulomatous Disease: A Comprehensive Update

**DOI:** 10.3390/biomedicines10102570

**Published:** 2022-10-14

**Authors:** Juan Agustín Garay, Juan Eduardo Silva, María Silvia Di Genaro, Roberto Carlos Davicino

**Affiliations:** 1 División de Inmunología, Facultad de Química, Bioquímica y Farmacia, Universidad Nacional de San Luis, San Luis 5700, Argentina; 2 Instituto Multidisciplinario de Investigaciones Biológicas (IMIBIO), Consejo Nacional de Investigaciones Científicas y Técnicas (CONICET), San Luis 5700, Argentina

**Keywords:** nitric oxide, disease, chronic granulomatous disease

## Abstract

Nitric oxide (NO), a signaling molecule, regulates multiple biological functions, including a variety of physiological and pathological processes. In this regard, NO participates in cutaneous inflammations, modulation of mitochondrial functions, vascular diseases, COVID-19, neurologic diseases, and obesity. It also mediates changes in the skeletal muscle function. Chronic granulomatous disease (CGD) is a primary immunodeficiency disorder characterized by the malfunction of phagocytes caused by mutations in some of the genes encoding subunits of the superoxide-generating phagocyte NADPH (NOX). The literature consulted shows that there is a relationship between the production of NO and the NADPH oxidase system, which regulates the persistence of NO in the medium. Nevertheless, the underlying mechanisms of the effects of NO on CGD remain unknown. In this paper, we briefly review the regulatory role of NO in CGD and its potential underlying mechanisms.

## 1. Introduction

Nitric oxide (NO) is an endogenous gaseous signaling molecule produced by Nitric Oxide Synthase (NOS) through the oxidation of L-arginine [1], which is highly active and mediates many physiological processes. Due to its chemical characteristic, NO diffuses freely across cell membranes, interacts with intracellular targets to activate signal transduction pathways, and plays different roles in biological systems [2], including vasodilation and signal transmission in neurons [3]. NO can also activate cellular and humoral immunity and has antibacterial properties. Additionally, it activates the proliferation of keratinocytes, the antioxidant system, and the proliferation and synthetic activity of fibroblasts [3].

Three isozymes of nitric oxide synthase (NOS) have been widely studied: endothelial nitric oxide synthase (eNOS), neuronal nitric oxide synthase (nNOS), and inducible nitric oxide synthase (iNOS) [4]. eNOS is mostly found in endothelial cells and is in charge of keeping the tone of the blood vessels. Numerous cell types, including neurons, heart muscle, and endothelial cells, contain its three primary isoforms. iNOS is typically located in macrophages [5] and can produce toxic amounts of NO, representing an important component in the antimicrobial, antiparasitic, and antineoplasic activity of these cells [4].

NO is a promiscuous signaling molecule with active participation in health and disease.

In this regard, its critical role in the modulation of inflammatory circuits in cutaneous tissue [6], the regulation of mitochondrial O_2_ consumption [7], the mediation of vascular relaxation through the second messenger cyclic guanosine monophosphate [1], and the adjustment of skeletal muscle contractile function have been demonstrated [8]. During SARS-CoV-2 infection, NO has played a protective role through four mechanisms: regulating blood flow, initiating anti-inflammatory responses, promoting anti-coagulation effects, and exerting antiviral properties [9]. Further, iNOS-derived NO can induce insulin resistance and glucose intolerance [10]. It is a well-known neuromodulator agent that participates in fear-like behavior [2], major depression pathogenesis [11], and memory consolidation processes exerting a context-dependent dual role [12,13]. On the other hand, NO is generated by almost all myocardial cell types and controls cardiac function through both vascular-dependent and -independent mechanisms [14]. It has been seen that the amount of NO in coronary heart disease is decreased [15] and that this could be due to a lower bioavailability of L-arginine [16]. In fact, there are therapies that restore optimal levels of NO to prevent heart failure [17].

Chronic granulomatous disease (CGD) is a hereditary illness in which phagocytic leukocytes fail to produce reactive oxygen species (ROS), such as superoxide anion (O_2_^−^) and antimicrobial oxidants. Catalase positive bacteria cause recurring infections in CGD patients [18]. It has been suggested that the CGD and NO are linked. In this regard, Tsuji et al. (2012) showed that polymorphonuclear neutrophils (PMNs) from CGD patients increase nitric oxide after phagocytes stimulation [18]. In this review, we focus on the current evidence that shows the intervention of NO in the physiopathology of CGD.

## 2. Chronic Granulomatous Disease (CGD) and NADPH Oxidase (NOX)

**Figure 1:**
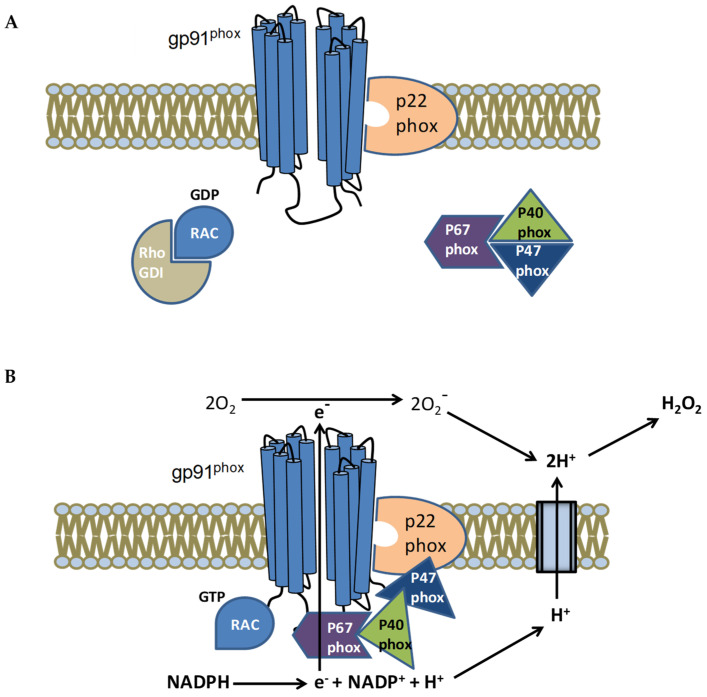
The NADPH oxidase (NOX) activation complex in the cytoplasmic membrane of a phagocyte is depicted in a schematic form. (**A**) The oxidase complex is inactive in the absence of stimuli, with gp91phox and p22phox attached to cell membranes and p67phox, p47phox, and p40phox in the cytosol. (**B**) The cytosolic p47phox subunit is phosphorylated in response to inflammatory stimuli, which activates NADPH oxidase and causes the assembly of all cytosolic components (p67phox, p40phox) to p22phox/gp91phox. Rac is also bound in conjunction with this. The active enzymatic complex moves electrons from the cytosol to phagosome lumen, where oxygen (O_2_) is changed into superoxide anion and then hydrogen peroxide (H_2_O_2_).

Chronic granulomatous disease is a primary immunodeficiency (PID) which affects 1 in 120,000–250,000 live births [19]. Patients with CGD present recurrent clinical manifestations [20] (Table 1). CGD is characterized by a defect in the bactericidal and fungicidal activity of phagocytes due to mutations in the enzyme complex nicotinamide adenine dinucleotide phosphate (NADPH) oxidase (NOX). This is an oxidase machinery that takes electrons from NADPH in the cytoplasm, generated by the hexose monophosphates hunt, and transfers them onto oxygen in the vacuole to produce O_2_^−^ [21] (Figure 1). The catalytic component of the phagocyte NADPH oxidase has six human homologs: NOX1, NOX3, NOX4, NOX5, DUOX1, and DUOX2. The homologs are collectively referred to as the NOX family of NADPH oxidases, together with the NOX2/gp91phox component found in the phagocyte NADPH oxidase assembly. NOX is a multidomain complex that requires different protein combinations for assembly in order to function [22].

## 3. Innate Immunity

The immune system has been traditionally classified into two categories: the adaptive immune system and the innate immune system [23]. Innate immunity consists of a series of physical, chemical, and anatomical barriers [24] that act as the first line of defense against all types of infectious agents, including extra [25] and intracellular [26] bacteria, viruses [27], fungi [28], protozoa [29], and helminths [30].

While adaptive immunity consists of two basic cell types, B lymphocytes and T lymphocytes, innate immunity has a more diverse cellular composition. In this regard, the innate immune system presents both hematopoietic and non-hematopoietic cells within the tissue barriers [31,32]. Innate hematopoietic cells are becoming important in health and disease [33]. There are several studies on innate immune cells of the myeloid lineage, being the most representative cells the neutrophils, eosinophils, basophils, mast, monocytes, macrophages, and dendritic cells [34].

The cellular components of innate immunity have a series of extra and intracellular molecules that allow an initial recognition of the pathogen [35]. In addition, they have a series of microstatic and microbicidal effector mechanisms to contain the infection during the first hours and days, thus triggering specific immune responses [33]. Therefore, innate immunity presents a series of humoral and cellular effector mechanisms. Humoral mechanisms include activation of the complement, coagulation cascade, lactoferrin secretion, and defensins [36]. Regarding cell-based effector mechanisms, phagocytosis together with cell-mediated cytotoxicity are predominant. Phagocytes are cells capable of perform phagocytosis, which sense a series of events triggered by the presence of molecular patterns associated with pathogens and/or molecular patterns associated with damage. The sequence of events includes migration, adhesion, diapedesis, and phagocytosis [36]. It is now acknowledged that phagocytosis is a cellular process that is not only involved in the immune response against pathogens but also in the preservation of homeostasis since it participates in the clearance of cell debris [37]. It is a highly regulated process favored by ligand-receptor recognition processes with subsequent engulfment of particles within the so-called phagosome [38]. The phagosome undergoes a series of maturation processes and drastic biochemical changes known as respiratory burst.

It is now recognized that innate immunity is not only a mere effector of adaptive immunity but also contributes to the optimization and course of the immune response by providing the appropriate cytokine microenvironment for the differentiation of T lymphocytes into a specific phenotype [39]. Cytokine networks established by innate immunity play a central role in the pathogenesis of various diseases with immunopathological bases [40]. The effectors and regulatory functions of innate immunity in immunodeficiency have also been studied. The condition known as CGD serves as a typical example (Primary Immunodeficiency) [41] which presents susceptibility to recurrent infections and the development of autoimmunity [42].

## 4. Immunomodulatory Properties of NO

NO has a variety of functions in immunity, including its role as immunoregulator, apoptosis modulator, and as toxic agent against infectious organisms. [43]. In this context, iNOS is the most relevant source of immunomodulatory NO, and its expression is upregulated through multiple proinflammatory signals [44,45] via NF-kB as a master inflammation regulator [46,47]. Despite their minor role, eNOS and nNOS may be important sources of NO at inflammation sites [48], and their expression is mediated by Ca^2+^ in response to multiple ligands [49].

Today, the microbicidal capacity of NO is well known [50,51] and many pathogens have developed immune response evasion mechanisms based on the inhibition of NO generation [52]. Thus, therapies based on NO-releasing agents are currently being developed to treat aggressive infections in humans [53].

In addition to its classic cytotoxic effects, NO plays a crucial role in the immune response regulation, establishing a link between innate immunity and adaptive immunity [54]. Experiments using iNOS-deficient mice showed that NO regulates adaptive immunity by restricting T cell proliferation, attenuating IFN-γ production, and differentiation to a Th1 phenotype [55], thus postulating NO as a self-regulation mediator [56]. Furthermore, NO is a potent immunoregulator in other T cell lineages, such as Th17 cells [57] and CD8(+) T cells [58]. Recently, the ability of NO to shape innate immune cell metabolic programs has been documented [59,60].

## 5. Relationship between NO and NADPH Oxidase

Both NOS and ROS species, generated by the concerted action of iNOS and NADPH oxidase, are known to play complementary roles in disease, such as progression of tumor growth [61,62], maintenance of intestinal bacterial homeostasis [63], microglial toxicity [64], or control of infections by opportunistic pathogens [65]. Furthermore, it has been shown that not only NO acts as a signaling molecule but that ROS- derived from NADPH oxidase- also has a regulatory function with associated signaling pathways [66,67]. It has been suggested that NADPH oxidase presents a higher hierarchy in the signaling of inflammatory circuits and that it controls the production of NO by modulating the expression of iNOS [67,68,69,70]. However, it has also been reported that iNOS activity is capable of regulating the function of the NADPH oxidase complex [71,72]. It seems that both enzymes influence each other, becoming more relevant depending on the context.

Given the demonstrated protective and regulatory role of NADPH oxidase [73,74,75], it is expected that patients with CGD present a complex series of immunopathological mechanisms besides immune deficiency. Patients with CGD showed an imbalance in their redox state with an increase in antioxidant activity, depletion of antioxidant metabolite levels, and higher lipoperoxidation scores together with a higher proportion of protein and nucleic acid oxidation products [76].

It has been largely reported that CGD patients can produce NO, so the activity of NOS isoforms is not completely dependent on the presence and activity of NADPH oxidase [77]. Thus, it has been shown that the NADPH oxidase system regulates the persistence of NO in the medium upon consumption, being the main enzymatic complex of phagocytes capable of regulating NO levels [78]. These findings suggest that CGD patients could present higher basal levels of NO or at least present problems in the regulation of its activity. Consistent with this, a spontaneous increase in NO production has been reported in in vitro cell models of CGD patients used as a negative control [79].

## 6. Impact of NO in the Pathophysiology of CGD

### 6.1. Susceptibility to Bacterial and Fungal Infections

CGD manifests with recurrent bacterial and fungal infections that can appear from infancy to adulthood. Males have been reported to be the most affected. The typical organs suffering from infections are the lungs, lymph nodes, skin, bones, and liver. In countries where the bacillus Calmette–Guerin (BCG) vaccine is routinely applied, the initial manifestation of CGD may be local or regional becegeitis [20]. Patients with CGD present a greater susceptibility to pyogenic and granulomatous infections, with a myriad of pathogens as possible causal agents [80]. In addition, the greater susceptibility to infections can not only be explained by the deficiency in the formation of ROS but particularly the neutrophils of patients with CGD present defects in the generation of NETs [21].

### 6.2. Granuloma Formation

CGD is a disease with the frequent formation of microscopic structures called granulomas. They are characterized by a predominance of macrophages transformed into epithelioid cells. Immune granulomas occur as a consequence of the development of an adaptive immune response, in which cellular immunity participates with the activation of TCD4^+^ Th1 lymphocytes (delayed hypersensitivity or type IV), which is induced in response to the presence of life-threatening intracellular pathogens [81]. Previous studies have found that granulomas derived from glycoantigens (e.g., *Staphylococcus aureus* capsule antigens) present in murine models of CGD are generated in a NO-dependent manner from dendritic cells. Interestingly, mice with CGD respond excessively to the presence of glycoantigens, generating granulomas via activation and proliferation of CD4+ T lymphocytes. This is because the overactivity of NO in dendritic cells facilitates the processing of glycoantigens by inducing deamination-depolymerization processes and their subsequent presentation under an MHC-II context (HLA-DM) [82]. On the other hand, it has been determined that dendritic cells from CGD patients fail to alkalinize their phagosomes and present problems in the cross-presentation of antigens due to excessive protein degradation [83]. Treatment of murine CGD models with 1400W, an iNOS inhibitor, not only attenuates NO production but also reduces the size and number of glycoantigen-induced granulomas in such models [82].

### 6.3. Chronic Inflammation

Several studies using three different murine models of CGD have elucidated that NOX-2 deficient mononuclear phagocytes are responsible for the hyperinflammation present in the disease. In addition, IL-1β has been shown to be the main pro-inflammatory cytokine released by these cells, and thus IL-1β antagonists could be used as anti-inflammatories in CGD patients [84]. The presence of high levels of IL-1β in patients with CGD implies the existence of factors that trigger the formation of the inflammasome required for the maturation and secretion of numerous proinflammatory cytokines, including IL-1β. ROS generation during the respiratory burst is one of the conventional signals required for inflammasome assembly, such as the NALP-3-like inflammasome. However, patients and murine models deficient in NADPH oxidase show activation of caspase-1 and secretion of IL-1β against inflammatory stimuli, indicating that a functional phagocyte oxidase is not essential in the inflammatory response of monocytes derived from CGD patients [85]. This implies that there could be other species generated during the respiratory burst that compensate or replace ROS in the assembly of the inflammasome. However, NO does not seem to be it, since previous studies have shown its inhibitory nature on the formation and function of the NALP-3 type inflammasome [86]. It is known that the activation of the autophagosomal pathway limits the activity of the inflammasome by ubiquitination and subsequent degradation [87]. Failure of the autophagy pathway to stop inflammasome activity has been suggested to be an essential component of diseases with chronic inflammation [88]. CGD is a disease with a significant prevalence of chronic inflammation with aberrant activity of the inflammasome and, paradoxically, with unbalanced NO production. As part of its numerous regulatory functions, NO can inhibit autophagosome formation and activity [89]. In this regard, the inhibitory effect of autophagy mediated by NO could predominate over its inhibitory effect on inflammasome activity, resulting in the generation of IL-1β, but studies are required.

### 6.4. Neurological Symptoms

Although neurological symptoms are not very frequent in CGD patients [90], neurological lesions such as demyelinating lesions, infiltrations of pigmented macrophages [91], vasculitis, hemorrhages, and infarcts in different neuronal structures [92] have been reported. Although it is recognized that inhibition of NADPH oxidase activity is involved in neuroprotective effects [72], it has also been acknowledged that it has a physiological role as a source of neuronal superoxide anion in response to the activation of the NMDA receptor (NMDAR), a glutamate receptor involved in processes of synaptic plasticity, learning, developmental plasticity, and neuronal death [93]. In a retrospective study of 26 CGD patients, 23% were found to have an IQ of 70 or less, indicating cognitive deficits [94]. In line with this, it has been shown that a NADPH oxidase deficiency is related to mild impairments in hippocampus-dependent memory, spatial memory deficit, and impaired context-dependent fear memory in murine CGD models [95].

It is well known that the activation of NMDARs induces the production of NO in the brain [96]. Thus, NO acts as a mediator of glutamate activator of the NMDARs in several nervous circuits, regulating processes such as hearing [97] and angiogenesis [98]. Interestingly, an absence of NADPH oxidase expression in different nerve centers as well as different degrees of impaired cognitive performance has been observed in nNOS-deficient mice [99], showing a relationship between both enzymes in cognitive processes. As has been proved, NO presents a well-established role as a vital mediator in the consolidation of memory and learning [100]. However, it has been reported that the inhibition of NO production has protective effects against memory and learning loss in specific pathological processes [13,101]. Even so, the benefits of the inhibition of NO production in memory processes and synaptic plasticity are due to the specific labeling of microglial or astrocyticic NOS [102]. Instead, the documented benefits of NO in cognition, learning, memory, and neurodevelopment appear to be mediated by neuronal nNOS in response to glutamate in long-term potentiation processes [103]. In addition, the correlation between nNOS activity and NMDAR activation is maintained in pathological processes such as Calcium-mediated excitotoxicity [72,104]. In the same process, it has been observed that NADPH oxidase inhibition prevents neuronal death and attenuates excitotoxic effects, suggesting a synergy in the activity of nNOS and NADPH oxidase [105,106]. However, more studies are required to explore the hierarchical relationship between both enzymes on the signaling pathways derived from the activation of NMDARs and its consequence in the synaptic plasticity of CGD patients.

### 6.5. Mechanisms of Hypersensitivity in Respiratory and Gastrointestinal Symptoms

Together with the susceptibility to the formation of granulomas present in CGD patients, through type IV hypersensitivity mechanisms, other clinical outcomes have been reported in these patients as a consequence of abnormalities in their immune system functions. A relationship has been found between hypersensitivity pneumonitis (HP) as an initial manifestation of CGD, especially in children [107,108,109]. The classification of HP as an interstitial lung disease describes it as an intricate immunological response of the lung parenchyma to repeated inhalation of a sensitized allergen. HP causes a combination of type-III and type-IV hypersensitivity reactions in the lung parenchyma. After initial sensitization, the offending antigen or chemical first induces a type III (immune complex-mediated) hypersensitive reaction. As long as the antigen is present, the reaction becomes a delayed (type IV) hypersensitivity reaction [110]. Interestingly, Shirai et al. (2010) described a 57-year-old male patient with HP, who presented alveolar NO concentration increased [111]. In addition, excessive NO production by alveolar macrophages plays a predominant role in lung damage due to oxidative stress in this disease [112]. Similarly, iNOS-derived NO plays an active role in the inflammatory processes of Crohn’s disease [113] and inflammatory bowel disease [114] both clinical presentations found in CGD [115,116].

### 6.6. Autoimmune Diseases

It is known that immunodeficiencies are related to autoimmune diseases in situations where deregulated immune responses against certain pathogens [117] occur. It has been reported that both autoimmune diseases and complications derived from an intense inflammatory state are more frequent in patients with CGD than in the rest of the population. In this regard, some findings suggest that the NADPH oxidase enzyme could be playing a critical role in the regulation of the adaptive immune response [117,118]. Thus, autoimmune diseases associated with CGD include discoid lupus, systemic lupus erythematosus, rheumatoid arthritis, idiopathic thrombocytopenic purpura [117], dermatomyositis, sacroiliitis, and autoimmune hepatitis [119], and the relationship between ROS and regulatory T responses is well known. Likewise, there is evidence suggesting a link between the ROS production and the induction of regulatory T (Treg) cells [120]. In this regard, Kraaij et al. (2010) showed that Treg cells can be induced by macrophages through a ROS-dependent mechanism [121]. Considering that Treg cells play a crucial role in the regulation of autoimmune responses [122] and that deficiency in ROS production is the hallmark of CGD, it is suggested that autoimmune diseases linked to CGD could be related, at least in part, to a decreased regulatory immune response associated with Treg cells. On the other hand, it is known that for the induction of Treg cells, interaction with an Antigen Presenting Cell (APC) is required [122]. Therefore, an impaired response of APC (macrophages and DCs) could be involved not only in the abnormal development of regulatory responses but also in the hyperinflammation state observed in both, CGD patients and animal models. However, the mechanisms by which the absence of ROS induces this failure in APC functions are still unclear. Additionally, results highlight the important role DCs play in inducing the CGD hyperinflammatory state, which could contribute to the development of autoimmunity. In this regard, Defert et al. (2012) demonstrated in CGD animal models that NOX2-deficient mice respond to intradermal injection with β-glucans showing high levels of proinflammatory cytokines (TNFα, IL-6, and IL1β) in the skin lesions. These cytokines were mainly secreted by macrophages and DCs [84]. It is known that DCs are critical actors in immune response, both, regulating the delicate balance between inflammation and tolerance and acting as linkers between innate and adaptive immunity [123,124,125]. Thus, there is a particular subset of NO and TNFα producing DCs (CD11b^+^ CD11c^int^-TIP DCs) which are derived from Ly6C^Hi^ monocytes and migrate to inflamed tissues [126,127]. On the other hand, Si et al. (2016) showed that DCs-derived NO controls the balance between the differentiation of effectors DCs and regulatory DCs. Thus, these authors reported that mice deficient in the NO-producing enzyme (iNOS) have an increased number of effectors DCs (IL-12, TNFα, and IL-6 producing), but a normal number of regulatory DCs (IL-10 producing) [123]. Therefore, NO would be acting as an inhibitor agent in the differentiation of effectors DCs. In this regard, the suppressive activity on NFκB pathways and inflammasome activation demonstrated that this molecule may contribute, at least in part, to the observed effects on DC differentiation [123]. These results demonstrate that DCs through NO plays a central role in the regulation of the immune response and in the avoidance of hyperinflammation states observed in CGD.

It is known that when apoptotic neutrophils cannot be phagocytosed by macrophages in an infectious focus, they can suffer necrosis and release their content into the environment, causing more inflammation and favoring autoimmunity [128]. Macrophages can recognize apoptotic neutrophils through the lipid phosphatidylserine (PS) and regulate the immune response by secreting TGFβ to control inflammation. There is evidence suggesting that ROS can induce apoptosis in neutrophils [129] and that both patients and mice with CGD have decreased/delayed exposure to PS. Therefore, it is hypothesized that the failed intake of apoptotic bodies present in granulomas could contribute to immunization with self-antigens and the development of autoimmunity [128,129].

Cahact et al. (2018) showed that both patients and mice with CGD present an alteration in the proportion of IgG isotypes, which was associated with an increased production of IFNγ and interpreted as a possible cause of the higher IgG2c production observed in B cells [130]. On the other hand, there are results showing that the defect in the NADPH oxidase enzyme could alter the repertoire of peptides presented by the MHCII molecule in B cells. These findings suggest that NADPH oxidase plays a critical role in the development of autoimmunity in CGD patients [117]. Therefore, the increased cytokines by DC and the participation of B cells could be the master key in the integration between increased T cell activation, antibody production, and development of autoimmunity related to CGD.

## 7. Therapeutic Considerations

Identification of the pathogenic variant(s) in one of the six genes that encode or permit assembly of the phagocyte NADPH oxidase subunits establishes the diagnosis of CGD. Pathogenic variants in CYBA, CYBC1, NCF1, NCF2, and NCF4 cause autosomal recessive CGD; pathogenic variants in CYBB cause X-linked CGD [131]. The phenotypic diagnosis of CGD is made by using the 1,2,3-dihydrorhodamine (DHR) test which evaluates the functionality of neutrophils by flow cytometry. The optimal therapeutic management of CGD is based on the antimicrobial prophylaxis, aggressive treatment of infectious and inflammatory complications, and in some cases, stem hematopoietic cell transplant [20] (Table 2). Currently, combination strategies that typically involve prophylactic antibacterial agents, antifungal agents, and immunomodulation via interferon-gamma (IFN-γ) are used [132]. In this regard, IFN-γ mediated therapy has been proposed to offer prophylactic benefits [133] promoting NO production. This, in turn may prevent bacterial-induced inflammation by depleting inflammasome activity [134]. Although there are discrepancies about whether or not IFN-γ therapy increases serum NO levels in CGD patients [77], many authors have found that prolonged IFN-γ treatment enhances the generation of NO through the activity of TNF-α [135]. It has been proposed that the increase in NO generation during the phagocytosis process generated by treatment with IFN-γ or Trimetropin-Sulfomethoxazole (used to treat bacterial infections) collaborates to achieve a more efficient respiratory burst [79,135] highlighting the aspect of NO as a molecular aggressor [136]. This role of NO is of particular importance in the immune response against *Mycobacterium tuberculosis* [137], one of the most frequent infectious agents in CGD patients [138]. In addition, IFN-γ treatment has been shown to enhance clearance of apoptotic bodies through a NO-dependent process in a CGD model of murine macrophages [128]. However, IFN-γ therapy has certain side effects such as fever, fatigue, myalgia, rash, erythema, and pain. The cost-benefit balance for the therapeutic use of IFN-γ is favorable, especially for patients with the X-linked variant and with a history of invasive aspergillosis. Despite the benefits, IFN-γ drug therapy does not prevent granuloma formation and does not appear to improve symptoms of chronic inflammation [139]. On the other hand, working with cells from CGD patients and murine models, it has been shown that the blockade of the IL-1β receptor restores autophagy and inhibits the activity of the inflammasome, generating beneficial effects such as the attenuation of inflammation, resistance to invasive aspergillosis, and improvement of symptoms typical of colitis [140]. Treatment with Anakinra, an IL-1β antagonist, showed pharmacological efficacy in the treatment of colitis in CGD patients. Rapamycin, an mTOR inhibitor and autophagy restorer, is capable of reducing the release of pro-inflammatory cytokines. Thus, it has been suggested that combination therapy with Anakinra and Rapamycin can be used to treat the inflammatory complications present in CGD patients [139].

## 8. Conclusions

Nitric oxide (NO) is a widespread gaseous mediator that acts through the activation of soluble guanylate cyclase or by inducing nitrosylation on different protein targets. Three isoforms of Nitric Oxide Synthase are the source of this signaling molecule, which acts as a neuromodulator, immunomodulatory, and regulator of cardiovascular tone in health and disease. In an immune context, NO originated by iNOS together with ROS generated by NADPH oxidase act as molecular aggressors. iNOS and NADPH oxidase have certain similarities. They are part of the effector mechanisms of phagocytes and both derived species have regulatory properties that shape the immune response with the transcription factor NF-kB. Different studies have evaluated whether the NO or the ROS of NADPH oxidase have a predominant role over the action of the other; however, to date, the results are inconclusive. Even though the expression of one of the enzymes is not dependent on the presence of the other, they are subjected to mutual influence. In this regard, in Chronic Granulomatous Disease (deficient NADPH oxidase), there is an unbalanced production of NO in response to inducing stimuli, such as IFN-γ. There are few but convincing works that demonstrate the participation of NO in the pathogenesis of CGD. Thus, the production of NO in phagocytes compensates for the ROS deficit in CGD patients treated with IFN-γ, increasing the quality of their respiratory burst and even improving other aspects of phagocytic function such as the clearance of apoptotic bodies. On the other hand, NO plays a pathological role in mediating the generation of granulomas in the presence of ubiquitous microbial components of a polysaccharide nature, one of the hallmark signs of CGD. In this regard, these patients present a series of less recognized features such as chronic inflammation, mucosal hypersensitivity reactions, autoimmune manifestations, and neurological symptoms. Given the pleiotropic effects of NO and its multiple functions, together with the critical regulatory functions of NADPH oxidase, it is likely that an unbalanced activity between both enzymes and their products plays a predominant role in the pathophysiology of these less conventional symptoms. Finally, it is known that one of the main pharmacological effects of IFN-γ is the increase in NO production, which acts as an executing arm of IFN-γ, mediating its beneficial and adverse effects in CGD patients. Thus, though the IFN-γ-induced NO production does not improve the number and size of granulomas, it seems that it promotes their formation. All in all, this review has addressed the pathophysiological aspects of NO and signaling ROS in CGD and highlighted the importance of a comprehensive knowledge of these mediators for the development of more rational therapies and the improvement of those already available.

## Figures and Tables

**Table 1:** Clinical manifestations of CGD.

Cutaneous Manifestations	Gastrointestinal Manifestations	Autoimmune Manifestations	Infections	Ophthalmic Manifestations
Photosensitive malar rash Discoid lupus erythematosus Recurrent aphthous Seborrheic dermatitis Infections Abscesses recurring on skin	Colitis/Diarrhea Inflammatory bowel disease Stomatitis Autoimmune hepatitis Granulomatous enteritis Recurrent liver infections Liver abscess	Lupus, Lupus-like síndrome Arthritis Oral ulcers Raynaud’s phenomenon IgA nephropathy	*Staphylococcus* *aureus* *Aspergillus fumigatus,* *Nocardia* *Burkholderia cepacia* *Serratia marcescens*	Chorioretinitis

**Table 2:** Clinical management of CGD.

Manifestations Treatment	Prevention of Primary Manifestations	Cure	Pregnancy Management
New azole drugs for fungal infections. Long courses of antibacterials. Abscesses may require percutaneous drainage or excisional surgery. Combination of antimicrobials and corticosteroids for inflammatory response	Antibacterials and antifungals combined with immunomodulatory therapy (IFN-ɣ).	Allogeneic hematopoietic stem cell transplantation (HSCT)	Trimethoprim, a folic acid antagonist, is discontinued during pregnancy. Sulfamethoxazole is typically administered. Data regarding the teratogenicity of itraconazole are limited.

## Data Availability

Not applicable.
